# Inorganic Nanocrystals Functionalized Mesoporous Silica Nanoparticles: Fabrication and Enhanced Bio-applications

**DOI:** 10.3389/fchem.2017.00118

**Published:** 2017-12-13

**Authors:** Tiancong Zhao, Nam-Trung Nguyen, Yang Xie, Xiaofei Sun, Qin Li, Xiaomin Li

**Affiliations:** ^1^State Key Laboratory of Molecular Engineering of Polymers, Department of Chemistry and Laboratory of Advanced Materials, Collaborative Innovation Center of Chemistry for Energy Materials (2011-iChEM), Fudan University, Shanghai, China; ^2^Queensland Micro- and Nanotechnology Centre, Griffith University, Brisbane, QLD, Australia; ^3^Department of Orthopedics, Changhai Hospital & Department of Spine Surgery, Changzheng Hospital, Second Military Medical University, Shanghai, China

**Keywords:** mesoporous, bioapplication, nanoparticles, core shell structures, asymmetric structure

## Abstract

Mesoporous SiO_2_ nanoparticles (MSNs) are one of the most promising materials for bio-related applications due to advantages such as good biocompatibility, tunable mesopores, and large pore volume. However, unlike the inorganic nanocrystals with abundant physical properties, MSNs alone lack functional features. Thus, they are not sufficiently suitable for bio-applications that require special functions. Consequently, MSNs are often functionalized by incorporating inorganic nanocrystals, which provide a wide range of intriguing properties. This review focuses on inorganic nanocrystals functionalized MSNs, both their fabrication and bio-applications. Some of the most utilized methods for coating mesoporous silica (mSiO_2_) on nanoparticles were summarized. Magnetic, fluorescence and photothermal inorganic nanocrystals functionalized MSNs were taken as examples to demonstrate the bio-applications. Furthermore, asymmetry of MSNs and their effects on functions were also highlighted.

## Introduction

In the past decades, the impressive advancement of nanotechnology has brought major progress in nano-related biological applications. Nano-size particles can serve as carriers for functional guest molecules, drugs, genes, etc. and effectively accumulate at infection site through the enhanced permeability and retention effect (EPR) (Matsumura and Maeda, 1986; Davis et al., 2008). They also serve as platforms for other *in-vivo* functions such as bio-sensing and imaging. Commonly reported nano-sized particles, liposomes, polymer, and dendrimers have emerged as fabulous platforms for *in-vivo* applications. Amongst those particles, mesoporous silica nanoparticles (MSNs) shall be one of the most promising materials for bio-related applications due to their good biocompatibility, tunable mesopores, large volume, simple and scalable fabrication process and easy functionalization (-NH_2_, -COOH, etc.). Large volume of works has been conducted by loading the mesopores of MSNs with drugs or other bioactive substances to avoid their degradation during blood circulation and raise delivery efficiency (Teng et al., 2015; Moller and Bein, 2017; Yang et al., 2017). Gatekeeper molecules were used to seal drugs within the pores, waiting for environmental changes at a specific *in-vivo* location, tumor for example, to achieve target and stimuli delivery (Fatieiev et al., 2015; Prasetyanto et al., 2016; Wu et al., 2016). Over the recent years, *in-vivo* distribution, toxicity, renal clearance, and other properties (He et al., 2011; Du et al., 2016; Chou et al., 2017) of MSNs have been studied thoroughly, making it one of the most understood and favored materials for bio-applications.

However, in contrast to inorganic nanocrystals with abundant optical, magnetic, electric, and other physical properties, MSNs possess no special features, serving merely as a slow release carrier for guest molecules, thus would be insufficient for performing bio-applications which require certain functions. Though loaded molecules can be used for functional applications, for example indocyanine green (ICG) (Li J. et al., 2013; You et al., 2017) and IR-820 (Zhao et al., 2017) for photothermal therapy (PTT), rose bengal (RB) (Lu et al., 2016) for photo dynamic therapy (PDT). Still, the rapid development of nanomedicine and other bio-applications, such as bio-sensing, drug delivery, bio-manufacturing/imaging, diagnosis and therapy, urgently demand for more functions such as magnetic, thermal, and fluorescence properties. Thus, MSNs are often functionalized by incorporating various inorganic nanocrystals, which provide a wide range of intriguing properties, but when used alone they would suffer from low biocompatibility and limited loading capacity. Upon the combination, either the original functions of inorganic nanocrystals are enhanced and more suited for *in-vivo* applications, or delicate design and employment of mesoporous silica (mSiO_2_) may bring brand new possibilities in functions. Numerous works have been done in this field, and many outstanding results have been achieved. The most commonly employed methods for incorporation would be the formation of core-shell structure, coating a layer of mSiO_2_ shell on inorganic nanocrystals. This review focuses on MSNs functionalized with inorganic nanocrystals, both their fabrication and bio-applications.

## Core/shell structure based inorganic nanocrystals functionalized mesoporous silica nanocomposites

Fabrication of mSiO_2_ can be traced back to 1992 (Kresge et al., 1992). Yet, although there have been large volume of works on the synthesis of ordered mSiO_2_ materials through EISA method (Zhao et al., 1998; Wan and Zhao, 2007), bulk materials are usually not suited for bio-applications. Fabrication of MSNs as well as coating of ordered mSiO_2_ onto single nanoparticles came later through sol-gel induced assemble of silane with surfactants, and many works have been done by various independent groups. For instance, Deng et al. fabricated core-shell structure with perpendicularly oriented mesoporous channels (Deng et al., 2008). After obtaining Fe_3_O_4_@dSiO_2_, a surfactant-templating approach using cetyltrimethylammonium bromide (CTAB) micelle as template was utilized for coating a uniform layer of CTAB/silica composite. mSiO_2_ shell with radial mesopores were acquired after extraction of CTAB. This core-shell structure with ordered mSiO_2_ shell have a uniform pore size of 2.3 nm, surface area of 365 m^2^/g and a pore volume of 0.29 cm^3^/g. These accessible uniform mesopores with high surface area and large pore volume are promising for many applications.

**Figure 1:**
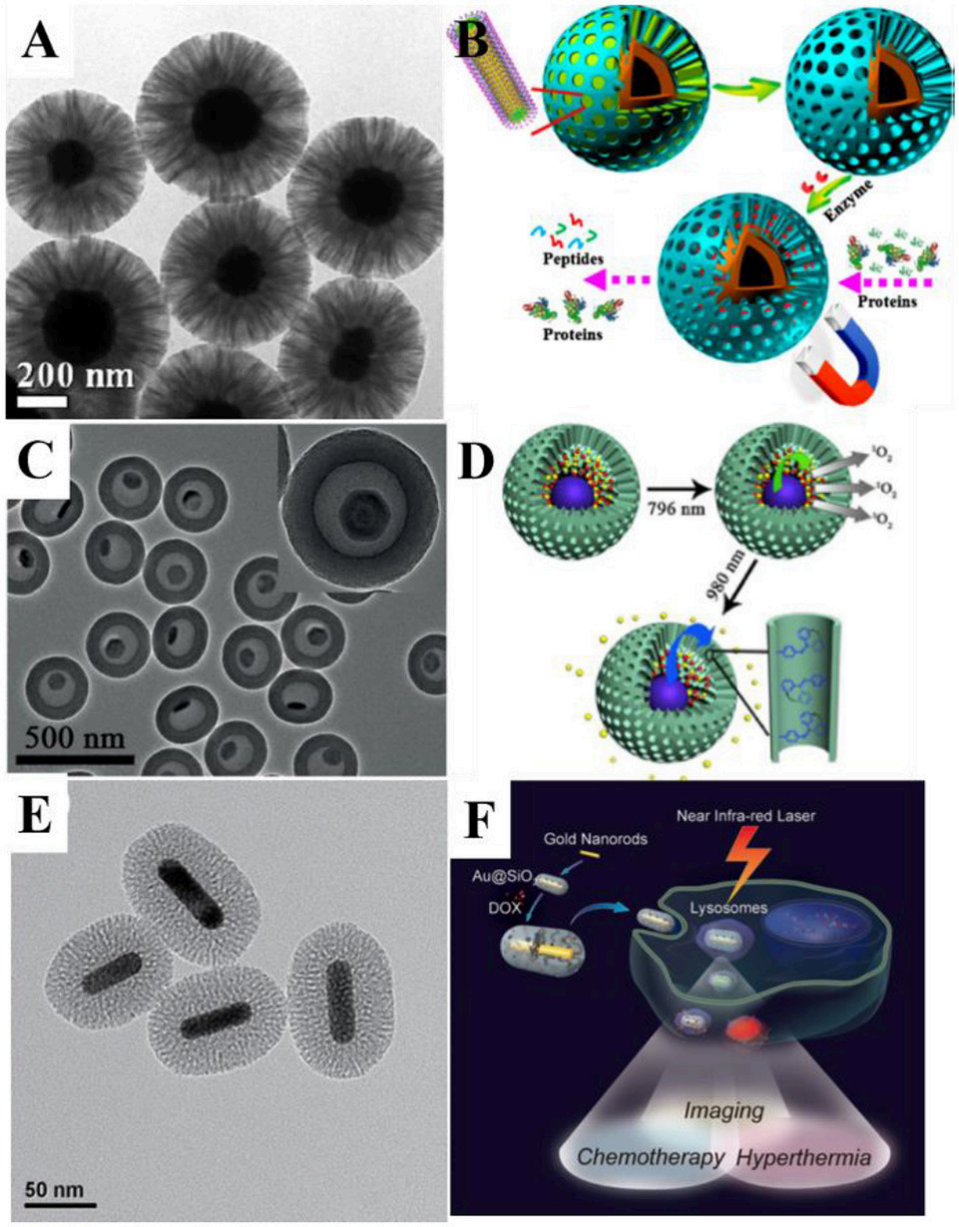
**(A)** Transmission electron microscope (TEM) image of Fe_3_O_4_@RF@mSiO_2_ nanoparticles and **(B)** application scheme of the nanocomposite for protein digestion. Reprinted with permission from Yue et al. (2015). Copyright (2015) American Chemical Society. **(C)** TEM image of the UCNPs@mSiO_2_ yolk-shell nanocomposites and **(D)** the scheme for combined photodynamic therapy and chemotherapy using 796 and 980-nm laser, respectively. Reprinted with permission from Li et al. (2016). Copyright 2016 Wiley-VCH Verlag GmbH & Co. KGaA, Weinheim. **(E)** TEM image of Au@mSiO_2_ nanoparticles and **(F)** schematic illustration of mSiO_2_-coated gold nanorods as a novel multifunctional theranostic platform for cancer treatment. Reprinted with permission from Zhang Z. J. et al. (2012). Copyright 2012 Wiley-VCH Verlag GmbH & Co. KGaA, Weinheim.

Although ordered mesoporous silica can be synthesized easily, it still suffers from the relatively small pore diameter of 2–3 nm, restricting loading guests to small molecules only, limiting its usages. Most recently, Zhao's Group proposed the bi-phase method, using silane and surfactants assembled at the water/oil interface to achieve expanded mesopores (Figures 1A,B; Yue et al., 2015). By changing different oil-phase (cyclohexane, octadecene, etc.) and the precise control of stirring speed, the growth of 3D-dendritic mSiO_2_ with pores as large as 15–20 nm can be achieved, greatly expanding the species and amount of potential loaded guest molecules. Such large-pore mesoporous silica coating may be used for loading of larger molecules, RNA, proteins, etc. (Shen et al., 2014; Xu et al., 2015; Yang et al., 2015; Meka et al., 2016; Zhang et al., 2016).

While water dispersible nanoparticles can be dispersed in water and ethanol, going through direct coating process, the growth of mSiO_2_ on oil-dispersed nanoparticles would be rather complicated. Nanoparticles fabricated through high-temperature solvent-thermal method often are capped with oleic acid (OA), oleyl amine, etc., which can only be dispersed in organic solvent such as cyclohexane, hexane, chloroform, etc. The previously mentioned method of coating mSiO_2_ can be adopted on OA-coordinated nanoparticles after their transfer to aqueous phase through reverse-emulsion method (Garcia et al., 2012; Jayakumar et al., 2012), a typical means for the coating of dense SiO_2_ on hydrophobic nanoparticles (Hu et al., 2009; Das et al., 2010; Jing et al., 2010). However, in order to form a stabilized emulsion, the amount of water in reverse-emulsion method is often limited to a very low level, resulting in rather low productivity, thus the method is not suitable for mass production. Another easier route is the direct coating of mSiO_2_, in which CTAB serves both as template for mesopore as well as stabilizer for water disperse. For example, Kim et al. used this method to synthesis core-shell structure with uniform morphologies such as Fe_3_O_4_@mSiO_2_, MnO@mSiO_2_ etc. (Kim et al., 2008).

By coating a layer of mSiO_2_ onto inorganic nanocrystals, their biocompatibility is significantly improved. In addition, the mesopores of mSiO_2_ can also provide a large surface area for loading vast amount of guest molecules, realizing functions such as diagnosis and therapy. Based on the above mentioned core/shell structure engineering, many kinds of inorganic nanoparticle@mSiO_2_ nanocomposites with different functions were fabricated and applied in bio-related applications.

### Magnetic nanoparticles functionalized mesoporous silica nanocomposites

Magnetic nanoparticles such as the popular Fe_3_O_4_ nanoparticles also include other forms of ferrites and particles doped with Gd^3+^, Mn^2+^, etc., possess potential biomedical applications in magnetic resonance imaging (MRI) (Kim et al., 2011; Hu and Zhao, 2012; Lee et al., 2014), and magnetic targeted drug delivery and separation (Giri et al., 2005; Gao et al., 2009; Sahoo et al., 2014; Wang and Gu, 2015). However, pure Fe_3_O_4_ (or other iron oxide) nanoparticles have a strong tendency of aggregating and are instable over long periods of internal cycles, limiting their *in-vivo* biological usages (Liu et al., 2011b). Thus, works on magnetic nanoparticles are often focused on their cooperation with mSiO_2_.

The cooperation of magnetic nanoparticles and mSiO_2_ results in multifunctional nanocomposites with both magnetic for MRI and mesopores for drug loading and delivery. Kim et al. achieved discrete encapsulation of one Fe_3_O_4_ nanoparticle in one MSN (Kim et al., 2008), the Fe_3_O_4_@mSiO_2_ core-shell nanoparticles with a diameter of 40–90 nm proved to be desirable T_2_ MR contrast agents. Zhao et al. synthesized rattle-type hollow magnetic mesoporous spheres with a cavity created through post hydrothermal treatment (Zhao et al., 2008), and set the nanocomposite as a drug loading and delivery model. As drug delivery nanocarriers, loading capacity is an important parameter, thus the pursue for large drug loading spaces becomes a quest. Although large pore volume may increase drug loading capacity, there's yet problems such as leakages, researchers seek to fabricated yolk-shell structure with interior vacancy. Chen et al. fabricated ellipsoidal Fe_3_O_4_@mSiO_2_ yolk-shell structure with a large cavity between the functional magnetic core and mesoporous shell (Chen et al., 2010b). In their work, SiO_2_ and mSiO_2_ were successively coated on the magnetic core, then the dense SiO_2_ layer was selectively etched, resulting in the formation of yolk-shell structure. The yolk-shell structured nanocomposite showed an excellent biocompatibility toward all kinds of cells. While the magnetic core served as an *in-vivo* monitor during the therapy as T_2_ contrast agent, the vacancy created proved to be an ideal drug loader with high DOX loading capacity. Such selective-etching methodology for creating controllable vacancy has been adopted by many other groups, utilizing the structure on various kinds of inorganic nanocrystals and expanding to applications even beyond drug delivery (Chen et al., 2010a; Zhu et al., 2010; Liu et al., 2011a; Wang et al., 2011).

Apart from magnetic resonance bio-imaging, another advantage of magnetic nanoparticles is the external magnetic field enhanced target drug delivery, a targeting method unnecessary for modifying of targeting molecules such as antibody (Kapse-Mistry et al., 2014). Liu et al. performed magnetic field directed cellular uptake experiment of Fe_3_O_4_@mSiO_2_ nanoparticles and achieved doubled uptake efficiency on A549 cancer cells (Liu et al., 2012). Xiong et al. fabricated Fe_3_O_4_@mSiO_2_ with large 15-nm radial mesopores for the delivery of siRNA (Xiong et al., 2016), a further layer of pH responsive tannic acid was deposed onto the nanoparticle to protect siRNA from degradation. Such design enabled magnetically induced high delivery efficiency and pH responsive release of siRNA. The enhanced gene delivery through magnetic vectors is given the name “magnetofection.” Much research has been conducted in the field, and showed promising future in *in-vivo* applications (Scherer et al., 2002; Mykhaylyk et al., 2007; Plank et al., 2011).

Current applications of magnetic nanoparticles functionalized MSNs are mostly focused on bio-imaging and targeted delivery of drug. Other functions such as detection and isolation (Song et al., 2011), magnetic hyperthermia (Wang D. W. et al., 2013; Yang et al., 2014), etc. might prefer more attention. Beside superparamagnetic nanoparticles, which at times face problems such as spin-canting, low saturation magnetization, and slow magnetic response, magnetic nanoparticles with vortex domain would also be a promising choice for bio-applications (Fan et al., 2010; Kim et al., 2010).

### Fluorescence nanoparticles functionalized mesoporous silica nanocomposites

Fluorescence nanoparticles find their usages in almost every field of bio-applications, including diagnosis, luminescence guided therapy (Lim, 2016), light triggered drug delivery, photodynamic therapy (PDT), etc. Quantum dots (QDs) and lanthanide doped upconversion nanoparticles (UCNPs) are the two most frequently used inorganic fluorescence nanoparticles (Li et al., 2013a,b; Li et al., 2014a, 2015a, 2016) However, most fluorescence nanoparticles suffer from bad aqueous dispersity due to the oil-phase solvent-thermal fabrication process, thus naturally leads to the coating of SiO_2_ to prevent aggregation and to protect the inner core from quenching or harsh environment (Li et al., 2015a).

Many works focused on fluorescence bio-imaging have been done. Li et al. designed CdTe@mSiO_2_ nanoparticles and realized real-time intracellular luminescence imaging of tumor cells (Li J. et al., 2014). Zhang et al. used NaYF_4_;Yb;Er@mSiO_2_ for cell imaging and drug delivery (Zhang et al., 2013). Lanthanide elements such as Gd^3+^, due to the complex 4f electrons, may also serve as MRI agents. Thus the same group proposed NaYF_4_;Yb;Er@NaGdF_4_;Yb core-shell UCNP encapsulated in mSiO_2_ for fluorescence and MRI dual-model bio-imaging (Li C. et al., 2013). Comparing to fluorescence imaging only, dual-model bio-imaging provide more accurate information toward the target, avoiding problems such as misdiagnose. Beside UV-vis fluorescence, second near-infrared window fluorescent bio-imaging of lanthanide doped nanoparticles also attracted a lot of attention. Both the excitation and emission are in the biological window for such infrared radiation (NIR) excited second NIR window (NIR-II) down-conversion fluorescent bio-imaging nanoparticles, allowing them to avoid many in-vivo disturbance, promoting the bio-imaging ability of the materials. For example, Wang fabricated SiO_2_-Nd@SiO_2_@mSiO_2_ nanoparticles with down-conversion fluorescence that can withstand harsh gastrointestinal conditions, serving as a real-time drug release monitor (Wang et al., 2017).

Stimuli-responsivity is of great significance in drug delivery, light triggered systems offer stimuli control both in time and location, thus would naturally attract attention. While various works have been done on controlled drug delivery under UV light, the near NIR triggering ability of UCNPs tend to be more valuable, since NIR radiation have better penetration and less damage toward tissues. Liu et al. contributed to the foundational work in the field (Liu et al., 2013). The group prepared NaYF_4_:Tm,Yb@NaYF_4_ core–shell nanoparticles, which emit UV/blue emission under NIR, and subsequently coated the UCNPs with mSiO_2_. “Photomechanical” azobenzene groups (azo) were then installed into the mesopores to act as UV/blue responsive “stirrer” to send out loaded guest molecules in the mSiO_2_. Such delicate design allows almost precise control of the amount of released DOX by varying the intensity or time of NIR light irradiation. This basic concepts were adopted by many other works that followed (Luo et al., 2013; Li W. et al., 2014; Min et al., 2014; Song and Yang, 2015).

Besides chemotherapy, photodynamic therapy (PDT) is another route for light-triggered therapeutic processes. PDT involves activation of photosensitizer (PS) with light to produce single oxygen, killing the tumor cells (Cui et al., 2013; Wang C. et al., 2013). Qian et al. incorporated zinc (II) phthalocyanine (ZnPc) into NaYF_4_;Yb/Er@mSiO_2_, using the NIR excited Er^3+^ green emission to active the photosensitizer to release reactive singlet oxygen for tumor therapy (Qian et al., 2009).

Loading of multiple guests and multifunctional fluorescence inorganic nanocrystals raise the possibility of designing functional MSNs with multiple controllable diagnosis and therapy process, thus the previously mentioned light triggering functionalities could be gathered altogether in one single nanoparticle. Combined therapy and diagnostic, theranostic process, allows more precise treatment toward disease. In order to realize imaging-guided combined PDT and chemotherapy, Li et al. fabricated six-layered UCNPs with orthogonal excitations-emissions upconversion luminescence and further combined them with mSiO_2_ to form the yolk-shell structured UCNPs@mSiO_2_ nanocomposites (Figures 1C,D). In this nanocomposite, the 1,050-nm NIR down-conversion luminescence (DCL) under low power density 796-nm excitation is used as a bioimaging signal. UV/blue emission under 980-nm excitation could be used to trigger the release of the chemotherapy drug in the hollow through introduction of azo groups. Independently, the PDT can also be realized under the stimulus of green emission excited by 796 nm (Li et al., 2016). Such design allowed the control of multiple therapy process through different excitation signal.

### Photothermal nanoparticles functionalized mesoporous silica nanocomposites

Many inorganic materials such as Au, CuS, graphene oxide, etc. can absorb and convert NIR light into thermal energy, causing localized heating which may effectively kill tumor cells. The therapy process of light triggered thermal effect is called photothermal therapy (PTT). Beside directly killing tumor cells through heat generation, the thermal energy can be used as a switch for drug delivery, either by heat-induced molecular movements or combining with thermal sensitive gatekeeper polymers. Yet, similar to other inorganic nanocrystals, this class of nanoparticles also suffer from low drug-loading capacity and easy aggregation.

In order to make up the above mentioned deficiencies, researchers try to combine photothermal materials with the mSiO_2_. Yang et al. synthesized cubic Ag@mSiO_2_ yolk shell structure, and then achieved Au nanocage@mSiO_2_ through galvanic replacement. A layer of thermal responsive polymer poly(nisopropylacrylamide) (PNIPAM) was coated outside the MSNs as a gatekeeper. Upon NIR radiation, heat generated by Au nanocages eventually lead to the collapse of PNIPAM shell, releasing the loaded DOX inside, realizing controlled PTT and chemotherapy (Yang et al., 2013). Such design prevented the leakage of drugs during *in-vivo* blood cycles, greatly raising the delivery efficiency. Zhang et al. coated mSiO_2_ with Au nanorods to used them as multifunctional theranostic platforms to serve as all-in-one two-photon imaging (TPI) agents, hyperthermia agent and drug carrier (Figures 1E,F; Zhang Z. J. et al., 2012). Other materials beside Au also possess photothermal ability, Chen et al. used CuS@mSiO_2_ as theranostic nanomedicine and ablated mice's 4T1 tumor after laser excitation with none regrowth over 2 months (Chen et al., 2015).

### Asymmetry mesoporous silica nanocomposites

Most recently, combined therapy with dual-drugs of different therapeutic effects showed excellent performance in treatment of diseases, especially in drug-resistant cancer treatments (Zhang et al., 2010; Zhang F. et al., 2012; Fan et al., 2013; Liu et al., 2015). One of the main challenges in realizing the best therapeutic effects is that the species and doses of drugs should be optimized at different clinical manifestations and periods in the treatment. However, simple drug delivery systems cannot fulfill the requirements of this combined therapy, because the widely used carriers such as MSNs (Deng et al., 2008), hollow structured nanoparticles (Zhang et al., 2016), yolk-shell nanoparticles (Zhu et al., 2010), etc. normally possess symmetrical geometry with limited single storage space available for loading of multiple drug species. When dual-drugs are loaded in a single storage space, release of each drug cannot be controlled independently. Therefore, developing asymmetric multi-compartment carriers with independent storage spaces for loading multiple drugs is critically desired. Nanoparticles with asymmetric nanostructures on geometry, chemical composition, surface property, and functionality, etc. have aroused extensive concern for their unique characteristics and potential applications. Compared with the conventional symmetric hybrid materials, the structural asymmetry is ideally suited for multiple-component conjugations, loading, or designing of smart delivery system on single-particle level. The nanocomposites with geometrical asymmetry determines that the function of each component can work independently without interaction with each other. These characteristics of the asymmetric nanoparticles render them truly “multifunctional entities.”

**Figure 2:**
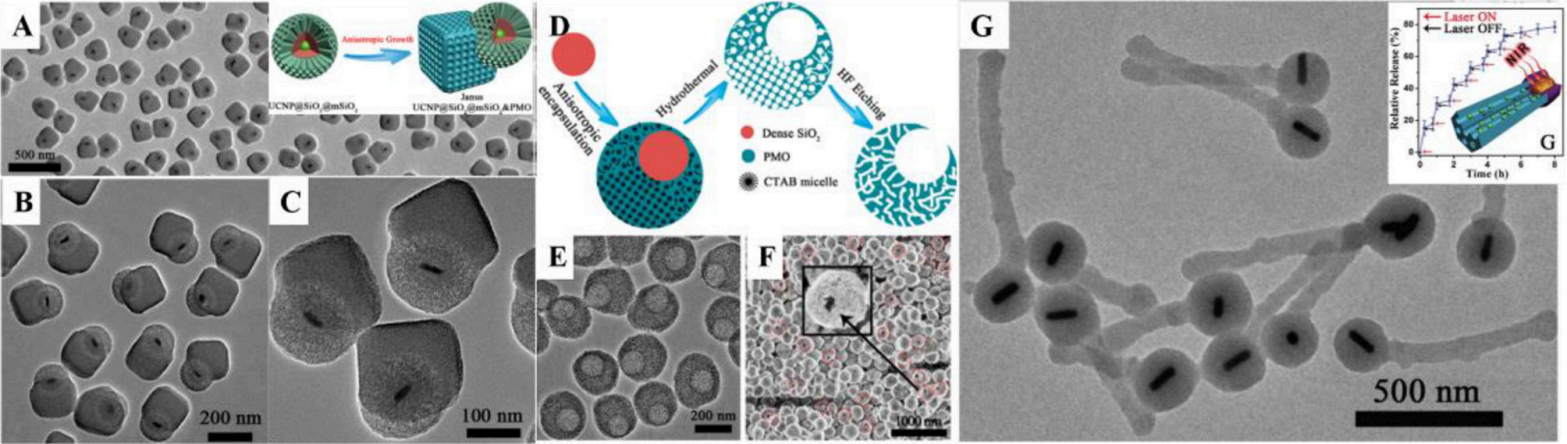
**(A–C)** TEM image of UCNP@SiO_2_@mSiO_2_&PMO Janus nanoparticles and the fabrication scheme (**A** inset). Reprinted with permission from Li et al. (2014b). Copyright (2014) American Chemical Society. **(D)** Scheme, **(E)** TEM, and **(F)** SEM images of asymmetry single-hole nanorattles. Reprinted with permission from Li et al. (2015b). Copyright (2015) American Chemical Society. **(G)** TEM image of match-like asymmetric Au-NR@SiO_2_&EPMO and the photothermal induced drug release (inset). Reprinted with permission from Li et al. (2017). Copyright 2017 Wiley-VCH Verlag GmbH & Co. KGaA, Weinheim.

Most recently, Li et al. first developed a novel anisotropic growth strategy for the fabrication of Janus structured dual-compartment multi-functional UCNP@SiO_2_@mSiO_2_&PMO (PMO = periodic mesoporous organosilica) nanocomposites. Start with the core@shell@shell structured UCNP@SiO_2_@mSiO_2_ nanospheres, the PMO single-crystal nanocubes can anisotropic island nucleation and growth on the side of the initial nanospheres to the unique Janus morphology (Figures 2A–C; Li et al., 2014b). The obtained nanocomposites are asymmetric not only in morphology, but also in pore size and hydrophobicity/hydrophilicity, which is much important for the multiple guests co-delivery. The distinct chemical properties of the silica sources and different mesostructures of the compartments are two key factors for the formation of the asymmetric nanocomposites. According to the specific designing of the switch at the entrance of the mesopores, the dual-compartment Janus mesoporous SiO_2_ nanocomposites are used in the dual-drugs co-loading and controllable releasing for high efficiency cancer cell killing. This synthetic strategy can be used in the fabrication of the asymmetry single-hole nanorattles for the co-delivery of dual guests with a great discrepancy in size (Figures 2D–F; Li et al., 2015b). Beside the anisotropic growth of cubic mesostructure, the same group also extended this strategy to the fabrication of 1D asymmetric di- and tri-block mesoporous nanocomposites with ordered hexagonal mesostructures (Figure 2G). Due to the 1D mesopore channels and the asymmetric in the function, the Au nanorod functionalized di-block Au-NR@SiO_2_&EPMO (EPMO = ethane bridged periodic mesoporous organosilica) nanorods exhibit superior controllable releasing of drug molecules triggered by near-infrared light (Li et al., 2017).

## Conclusion and perspectives

Functional mesoporous nanoparticles with symmetric and asymmetric architectures possess both unique properties of ordered mesoporous materials and abundant optical, electrical, magnetic properties of inorganic nanomaterials, showing great potential applications on catalysis, adsorption, separation, especially in biology applications.

Despite achieving enormous delightful results, there is still much room for further development. First of all, although many reported works have done intracellular experiments of the fabricated functional MSNs, their *in-vivo* performances still need further assessment. After incorporation of inorganic nanocrystals, long-term toxicity and pharmacokinetics of the materials used for *in-vivo* applications require careful monitor, so does biodegradation and side-effects, to ensure bio-safety of MSNs. Future works on functional MSNs call for more thorough design with consideration for not only *in-vitro* but also *in-vivo* experiments as well.

Due to the complexity of biological systems, single-functioned nanoparticles often fail to fulfill their assignments, leading to problems such as the danger of misdiagnose and resistance to drugs. Thus, the design and fabrication of multifunctional MSNs would be of great importance for future research. In current fabrications of functional MSNs, mSiO_2_ mainly serve to improve biocompatibility and load drug molecules, so that the properties of inorganic nanocrystals and MSNs are often isolated. The synergy between the inorganic nanocrystals and mSiO_2_ should be considered not only in the synthesis but also in the applications.

In many recent works, researchers are not satisfied by only controlling the pore size and overall diameter of MSNs. They seek to precisely tune the configuration of the functional MSNs, surface roughness for example, to render the nanocomposites brand new properties. Such investigations may bring new development to this field.

Furthermore, it still remains a major challenge to prepare MSNs with controlled loading and release of multiple loaded guest molecules, which would be essential for complex theranostic process. Such quest may be achieved through designing asymmetric multi-compartment MSNs and multifunctional inorganic core. While nanocomposites with geometrical asymmetry ensure the function of each component to work independently, multifunctional inorganic core may provide each compartment with the needed stimuli for controlled theranostic process, lifting the properties of functional MSNs to a new level.

In conclusion, inorganic nanocrystals functionalized mSiO_2_ nanomaterials will continue to be a hotspot and research focus. Further investigations into the system would beyond doubt bring exciting achievements and lead to promising clinic and other bio-applications in the near future.

## Author contributions

All authors listed have made a substantial, direct and intellectual contribution to the work, and approved it for publication.

### Conflict of interest statement

The authors declare that the research was conducted in the absence of any commercial or financial relationships that could be construed as a potential conflict of interest.
